# Mild stimulation approach for In Vitro Fertilization treatment: Retrospective data from one Danish Centre

**Published:** 2018-06

**Authors:** G Almind, E Faerch, F Lindenberg, S Lindenberg

**Affiliations:** Copenhagen Fertility Center, Lygten 3c, 2400 Copenhagen, Denmark

**Keywords:** Ovarian stimulation, IVF, ART, low dose stimulation, mild stimulation, minimal stimulation

## Abstract

**Aim of study:**

Over the last decade, the laboratory procedures in artificial reproduction have improved. Hyperstimulation causes an overload of eggs which will never be used. The present study was designed to evaluate the efficiency of a mild stimulation. To obtain oocytes for In Vitro Fertilization (IVF) a short antagonist protocol using Tamoxiphene and FSH was compared to conventional IVF.

**Methods:**

A retrospective and observatory study including all patients with unexplained infertility. In total 720 cycles with mild stimulation protocol and 8,446 cycles with regular short antagonist IVF protocol were analysed. The observation period was from January 2011 until September 2017. All patients were recruited in the same time period and allocated to different treatments upon their request. Low stimulation using orally administrated anti-estrogenic drugs combined with FSH in the form of injections was used in order to obtain up to four mature follicles.

**Results:**

The clinical pregnancy rate (CPR) per embryo transfer (ET) was 25% for the mild stimulation group. The CPR for the control group with conventional IVF was 23%.

**Conclusion:**

Mild stimulation may be an important step towards an easier IVF approach, more tolerable for women, easier and cheaper for the women and the society, while maintaining an acceptable success rate in terms of CPR. Large prospective studies need to be performed.

## Introduction

Affordable artificial reproductive techniques (ART) with low dose stimulation provide a safer and more acceptable treatment for most women undergoing fresh in vitro fertilization (IVF) treatment cycles because they cause less stress and are more convenient for the patient (Nargund et al., 2018). Recent data about embryo quality from mild/natural stimulation indicate a better implantation rate for embryos deriving from mild/natural stimulation compared to regular stimulation IVF (Farquhar et al., 2017; Martin et al., 2018). Gameiro et al. found physical or psychological stress to be the most common causes of discontinuation of fertility treatment in a review (Gameiro et al., 2012).

Old types of oral anti-estrogenic medicine such as Tamoxiphene and Clomiphene Citrate (CC) have been re-introduced in assisted reproduction for several reasons of which the primary reason is safety for the patient (Branigan et al., 2000). The evidence from a Cochrane review suggests that use of CC along with gonadotropins for ovarian stimulation leads to similar live birth rate per women when compared to gonadotropins alone (Gibreel et al., 2012). Inclusion of oral agents decreases the total gonadotropin requirement, reduces the risk of ovarian hyperstimulation syndrome (OHSS), and reduces the cost of medication (Lindenberg et al., 2013; Kamath et al., 2017).

Tamoxiphene blocks the spontaneous LH surge sufficiently when administrated during the whole length of stimulation, avoiding the need for an antagonist and making everything easier for the patient (Lindenberg et al., 2013). Thus, Tamoxiphene provides a simple dual endocrine modulation by stimulating follicular growth and blocking the LH surge. Mild stimulation could be less stressful for the patient and the medication more convenient.

The aim of this study was to evaluate the efficiency of mild stimulation with Tamoxiphene and FSH when compared with our routine IVF treatment using a short antagonist protocol.

## Materials and methods

We performed a retrospective cohort study including all patients admitted to IVF for unexplained infertility, aged 19-45 (mean age 35). Male and or tubal factors were excluded. All patients were recruited in the same time period and allocated to the different treatments on their own request. In total the study covers 720 cycles with a mild stimulation protocol and 8,446 cycles with a regular short antagonist protocol.

The study was conducted between January 2011 and September 2017.

### Mild stimulation protocol

In the mild stimulation protocol, we used orally administrated anti-estrogenic drugs, Tamoxiphene 20 mg or 40 mg daily depending of antral follicle count (AFC). 20 mg Tamoxiphene was used if AFC was seven or more and 40 mg Tamoxiphene was used if AFC was less than seven. The dose was administrated orally from day three in the cycle until the day before inducing final oocyte maturation. Human menopausal gonadotropin (hMG) or recombinant FSH was added in the form of injections (50 to 150 IU/every other day) depending on AFC in order to obtain up to 4 mature follicles. Monitoring ultrasound was initiated on day three and continued until follicles of 17 mm were seen. Then ovulation induction using Ovitrelle (Merck), human choriogonadotropin (hCG) was performed. Oocyte retrieval was done 34 hours after the trigger. Fresh embryo transfer (ET) was done on day two. A pregnancy test (urine hCG) was carried out 14 days after the oocyte retrieval date. Clinical pregnancy was confirmed in the 7th gestational week with presence of a gestational sac and positive heartbeat.

### Conventional stimulation protocol

The conventional short antagonist protocol included 150-400 IU FSH injections daily from day three in the cycle until the follicles had reached 17 mm in diameter. At this point a human choriongonadotropin (hCG) was given to induce final maturation (Ovitrelle, Merck). From the 6th day of FSH stimulation 0.25 mg Orgalutran (SUN Pharma) was administrated daily to avoid premature ovulation. Monitoring ultrasound scans were initiated at day three in the cycle and hereafter on day nine and on the day of ovulation induction.

Only treatment cycles with fresh ET were analysed.

## Results

A total of 720 mild IVF cycles were carried out using a mild stimulation protocol and 8,446 cycles in the control group.

A proportion of cycles were cancelled either prior or after oocyte retrieval in both groups (Table I). In the mild stimulation group 260 cycles (36%) were cancelled and a total of 460 cycles had fresh ET. For the conventional IVF group 2,005 (24%) got cancelled and 6,441 cycles had fresh ET and no significant differences were seen using t-test.

**Table I:** Proportion of cycles cancelled and causes of cancellations in the mild stimulation protocol (720 cases) and in the control group (8446 cases). No significant differences were seen.

Cancellations of all cycles started	Number of cycles cancelled	Before oocyte retrieval	Oocyte retrieval	After oocyte retrieval	
		Zero follicular development	Zero embryos at oocyte retrieval	Zero fertilisation	Zero embryos for transfer
Mild stimulation 36%	260	6,8%	7,7%	15,7%	5,8%
Control IVF 24%	2005	5,5%	3,4%	9,9%	4,8%

The pregnancy rate per transfer was equal between the protocols. Clinical pregnancy rate (CPR) after ET was 25% and 23% for the mild stimulation group and control group respectively (Table II). No significant differences were seen on an unpaired t-test comparing the CPR in percent between the mild group and the control group aged 19-39 (p = 0.0604) and aged 40-45 (p = 0.7545) (Table II).

**Table II:** Admitted patients, cancellations, embryo transfer (ET) and clinical pregnancy rate (CRP) pr. ET in the mild stimulation group and the control group in the whole group 19-45 years of age and in the subgroups 19-39 years of age and 40-45 years of age.

	Admitted patients	Cancelled Number/%	ET Number	CPR pr ET 19-45yrs number/%	CPR pr ET 19-39yrs number/%	CPR pr ET 40-45yrs number/%
Mild stimmulation	720	260/36 %	460	115/25%	93/29%	22/16%
Regular IVF	8446	2005/24%	6441	1475/23%	1041/32%	434/13%

**Figure 1:**
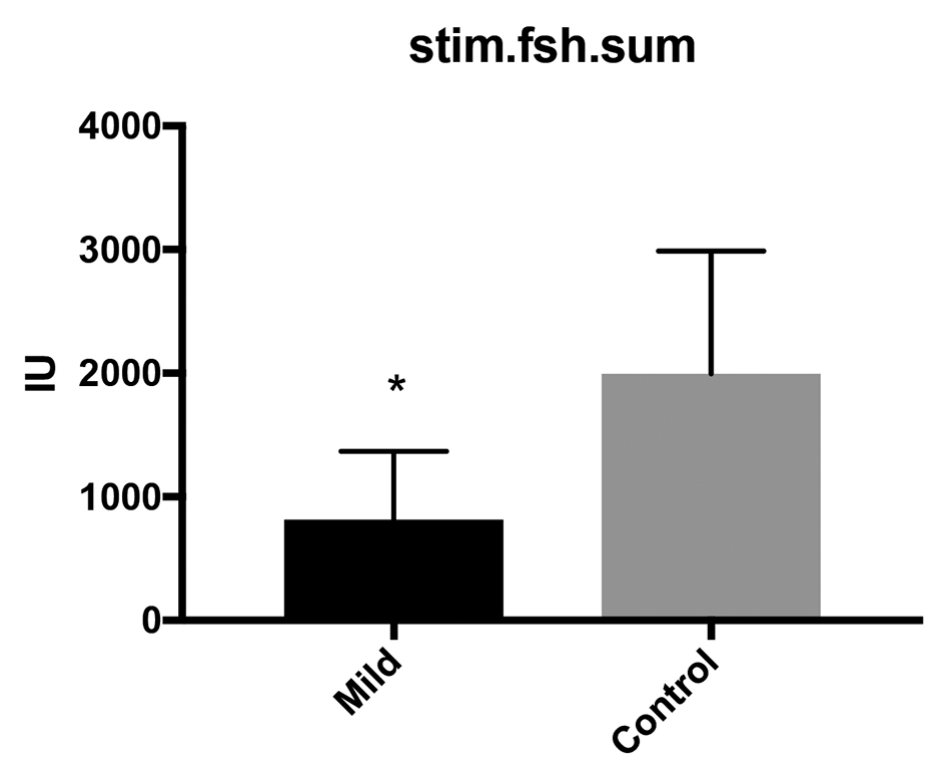
Differences in FSH units for the mild stimulation protocol compared to the control group.

The mean number of FSH units used in the mild stimulation group was 816.3 IU per cycle of women with positive urine HCG and for the conventional group a mean number of 1996 IU was used. Significant differences were seen on a F test to compare variances (p<0.0001) (Figure 1).

The average number of eggs retrieved in the mild stimulation group was 3.3 and the average number of embryos transferred was 1.4 embryos. For the conventional IVF group, the average number of eggs retrieved was 5.5 and average number transferred was 1.7.

## Discussion

The present retrospective cohort study demonstrates that the clinical outcome from the mild stimulation protocol was comparable with the control IVF protocol with a trend for better results in the group above 40 years of age in the mild stimulation group. However, the cycles numbers are low in this category with only 22 cycles. Not surprisingly and in line with previous studies an age-related decline in success rates was also demonstrated in both groups.

There is on-going scepticism among clinicians concerning the level of pregnancy rates in mild stimulation treatment due to less oocytes and subsequently an increased risk of cycle cancellation. In the present study, we did see cancellation due to lack of follicles and due to insecurity especially in the early years of using the mild stimulation protocol. Overall 19% of cancellations were due to lack of follicles or too few follicles. Initially the assumptions about the required numbers of follicles were too high in the clinic. As a consequence, more mild stimulation cycles were cancelled prior to egg retrieval in the early years.

Conventional IVF aims to retrieve a large number of oocytes to maximize the number of embryos available for ET or cryopreservation. Van der Gaast reported the optimal number of oocytes associated with the optimal chance of conceiving after ET to be 13 (van der Gaast et al., 2006). The patients going for mild stimulation protocol need to be informed properly about the aim for few follicles. In Scandinavia, elective single embryo transfer (eSET) has been practiced for many years and as a consequence it is unnecessary to harvest more than a few eggs in one IVF cycle. SET combined with mild stimulation may be an important step towards a less aggressive and more affordable approach in IVF. Other randomized studies found a trend toward a higher proportion of good-quality embryos/ blastocysts when using low stimulation protocols (Casano et al., 2012; Hohmann et al., 2003). A better quality of eggs in mild stimulation might account for the equivalent CPR when comparing mild and conventional IVF.

Advantages with this approach include more tolerable, cost efficient and problem-free treatment for the women while maintaining an acceptable effectiveness in terms of CPR. In addition, the reduction in multiple gestation rates can be mentioned. The embryo wastage in regular IVF can be reduced and can be more attractive in countries with strict legislation where destruction of surplus embryos, both fresh and frozen is forbidden.

Regarding ovarian hyperstimulation syndrome (OHSS) the mild stimulation protocol could be an option for all patients and in particular the women at risk of OHSS. Kato et al. (2012) did not observe any OHSS in their minimal/natural cycle IVF protocol. In the present study, four women were cancelled (1.5%) due to risk of OHSS compared to the control group where 37 were cancelled (2%). In our clinic, we always use the mild stimulation for women at risk of OHSS.

Two meta-analyses reported lower ongoing pregnancy rates with low stimulation compared to conventional IVF (Matsaseng et al., 2013; Verberg et al., 2009). Another meta-analysis of ten randomised controlled trials (RCT) found no differences in pregnancy rates. And in a recent review, 20 RCT were identified and all but two claimed mild stimulation to be equal to conventional IVF in pregnancy rate per ET (Nargund et al., 2018). Results from a large study with more than 20,000 cycles from minimal/natural stimulation show a similar level of pregnancy rates when compared to conventional IVF treatment (Kato et al., 2012).

Overall, this study suggested that extended Tamoxiphene use along with low-dose gonadotropin could be a mild stimulation IVF option.

However, limitations of the present study are related to the relatively small number of cycles and the retrospective nature of the study. Large prospective studies need to be performed to give the final answer whether mild ovarian stimulation protocols will become the first line option in IVF programmes.

## Conclusion

In conclusion, the clinical pregnancy rates per ET were comparable between the mild and conventional stimulation protocol for IVF in our centre.

Furthermore, mild stimulation using Tamoxiphene and low-dose gonadotropins seems to be a low risk and low-cost option in an IVF programme and could become the first choice of treatment for many patients in the future.

## Conflicts of interest:

None of the authors has reported any conflicts of interest. Any external funding did not support this study.
